# In Vitro Effectiveness of Microspheres Based on Silk Sericin and *Chlorella vulgaris* or *Arthrospira platensis* for Wound Healing Applications

**DOI:** 10.3390/ma10090983

**Published:** 2017-08-23

**Authors:** Elia Bari, Carla Renata Arciola, Barbara Vigani, Barbara Crivelli, Paola Moro, Giorgio Marrubini, Milena Sorrenti, Laura Catenacci, Giovanna Bruni, Theodora Chlapanidas, Enrico Lucarelli, Sara Perteghella, Maria Luisa Torre

**Affiliations:** 1 Department of Drug Sciences, University of Pavia, Viale Taramelli 12, 27100 Pavia, Italy; elia.bari@unipv.it (E.B.); barbara.vigani@unipv.it (B.V.); barbara.crivelli@unipv.it (B.C.); paola.moro@unipv.it (P.M.); giorgio.marrubini@unipv.it (G.M.); milena.sorrenti@unipv.it (M.S.); laura.catenacci@unipv.it (L.C.); theodora.chlapanidas@unipv.it (T.C.); marina.torre@unipv.it (M.L.T.); 2 Research Unit on Implant Infections, Rizzoli Orthopaedic Institute of Bologna, Via di Barbiano 1/10, 40136 Bologna, Italy; carlarenata.arciola@ior.it; 3 Department of Experimental, University of Bologna, Diagnostic and Specialty Medicine (DIMES), Via San Giacomo 14, 40126 Bologna, Italy; 4 Department of Chemistry, University of Pavia, Viale Taramelli 16, 27100 Pavia, Italy; giovanna.bruni@unipv.it; 5 Osteoarticular Regeneration Laboratory, Rizzoli Orthopaedic Institute of Bologna, Via Giulio Cesare Pupilli 1, 40136 Bologna, Italy; enrico.lucarelli@ior.it

**Keywords:** silk sericin, *Chlorella vulgaris*, *Arthrospira platensis*, spray drying, microspheres, wound healing

## Abstract

Some natural compounds have recently been widely employed in wound healing applications due to their biological properties. One such compound is sericin, which is produced by *Bombix mori*, while active polyphenols, polysaccharides and proteins are synthetized by *Chlorella vulgaris* and *Arthrospira platensis* microalgae. Our hypothesis was that sericin, as an optimal bioactive polymeric carrier for microencapsulation process, could also improve the regenerative effect of the microalgae. A solvent-free extraction method and spray drying technique were combined to obtain five formulations, based on algal extracts (*C. vulgaris* and *A. platensis*, Chl and Art, respectively) or silk sericin (Ser) or their mixtures (Chl-Ser and Art-Ser). The spray drying was a suitable method to produce microspheres with similar dimensions, characterized by collapsed morphology with a rough surface. Art and Art-Ser showed higher antioxidant properties than other formulations. All microspheres resulted in cytocompatibility on fibroblasts until 1.25 mg/mL and promoted cell migration and the complete wound closure; this positive effect was further highlighted after treatment with Art and Art-Ser. To our surprize the combination of sericin to Art did not improve the microalgae extract efficacy, at least in our experimental conditions.

## 1. Introduction

Bioactive compounds from natural matrices are valuable and attractive products for the pharmaceutical and cosmetic industry [1,2]. Microalgae are unicellular microorganisms, which react to the harsh conditions of their habitats (temperature, salinity, light intensity) with adaptive mechanisms, such as the synthesis of a wide range of biologically active secondary metabolites [3]. The different metabolic pathways activated as defense strategies by each species of microalgae explain their immense diversity in terms of structural and chemical composition [4]. Several studies demonstrated the antioxidant [5], anti-inflammatory [6] and antimicrobial [7] properties of microalgae due to their content in polyunsaturated acids, vitamins, pigments, polyphenols, polysaccharides and proteins [8]. 

*Arthrospira platensis* and *Chlorella vulgaris*, approved by the Food and Drug Administration (FDA) as GRAS (Generally Recognized As Safe), are two of the most interesting and abundant species of microalgae, with an annual industrial production of 3000 and 4000 tons, respectively [9]. These photosynthetic organisms, generally cultivated in open ponds, rapidly grow under extreme environmental conditions: the simple and low-cost cultivation systems make *A. platensis* and *C. vulgaris* suitable for industrial-scale production [10]. Over the years, several studies have been performed on both microalgae species to optimize the extraction and purification of bioactive compounds, potentially useful for different medicinal applications [11]. 

*A. platensis*, also called Spirulina due to its helical filamentous morphology, is a Cyanobacterium rich in proteins (60–70% *w*/*w*), vitamins (4% *w*/*w*, particularly pro-vitamin A, B_1_, B_2_, B_6_, B_12_, E and D), polysaccharides, carotenoids, minerals and essential fatty acids [12]. Due to its chemical composition, *A. platensis* has been extensively used as a nutritional supplement [13] and, nowadays, it is also considered a potential pharmaceutical source due to its antioxidant, immunomodulatory and anti-inflammatory properties, as demonstrated both in vitro and in vivo [14]. Multiple health and nutritional needs have also been met by *Chlorella vulgaris* extracts, for which its essential amino acid, vitamin, fatty acid and mineral contents is known for and has been reported [15]. Moreover, *C. vulgaris* contains several polysaccharides, including β-1,3-glucan, an active immunostimulator and a free-radical scavenger, [16] and a significant amount of antioxidant pigments, such as β-carotene, lutein and chlorophylls [17].

In cosmetics and in wound healing dressings, sericin has been appreciated as an additive due to its biological activities [18,19,20]. Silk sericin has long been known to be a potent natural antioxidant due to the high content of hydroxyl amino acids [21]. It has been reported that silk sericin exhibits ROS (Reactive Oxygen Species)-scavenging, anti-tyrosinase, anti-elastase, immunomodulatory activities [22] and promotes wound healing and collagen deposition by fibroblasts and keratinocytes [23].

Silk sericin for wound healing has been investigated in two clinical trials (www.clinicaltrials.gov): a wound dressing composed of silk sericin/PVA was shown to accelerate the healing of split-thickness skin graft donor sites as compared to the commercially available Bactigras^®^ (ID. NCT02091076, study completed). In another study (NCT01539980, study completed), the application of a silver zinc sulfadiazine cream, with or without silk sericin powder, confirmed the efficacy of sericin in promoting the re-epithelialization of a second degree burn in 5–7 days less than in the control group.

In a previous paper, we demonstrated that the biological effect of a natural flavanone, such as naringenin, was surprisingly increased when associated with sericin [24]. Based on this evidence, we hypothesized that sericin could improve the regenerative effect of the microalgae extracts. To the best of our knowledge, the association of silk sericin and microalgae aqueous extracts has never been tested in wound healing fields: in the present work, these natural bioactive compounds have been selected to assess their synergic effects on cell proliferation and migration. The aqueous extracts of both *C. vulgaris* and *A. platensis* were combined with silk sericin to manufacture microspheres intended for the topical treatment of skin wounds, and a set of characterization analyses has been carried out to verify the final product performances.

## 2. Methods

### 2.1. Materials

Bovine serum albumin (BSA), phenol, glucose monohydrate, sulfuric acid 99.999%, 2,2-diphenyl-2-picrylhydrazyl hydrate (DPPH), 3-(4,5-dimethylthiazol-2-yl)-2,5-diphenyltetrazolium bromide (MTT) and dimethyl sulfoxide (DMSO) were purchased from Sigma-Aldrich (Milan, Italy). Human fibroblast cells were obtained from the European Collection of Authenticated Cell Cultures Cell Bank (ECACC, Salisbury, UK), while all reagents used for cell cultures were purchased from Euroclone (Milan, Italy).

### 2.2. Preparation of Microalgae Extracts

*Chlorella vulgaris* and *Arthrospira platensis* were purchased from Archimede Ricerche S.r.l (Camporosso, Imola, Italy). Microalgae were added to distilled water (2.5% *w*/*v*) and heated at 105 °C for 15 min in autoclave (Auclave 760, Asal Srl, Milan, Italy). After cooling, microalgae suspensions were centrifuged at 3000× *g* for 10 min and the algal aqueous extracts [25] were collected. Known volumes of both extracts were dried in a stove to calculate the final concentrations. 

### 2.3. Extraction of Silk Sericin

The sericin extraction was performed as previously reported [22]: briefly, *Bombyx mori* cocoons were cut, soaked in distilled water (40 mL/g of cocoons) and autoclaved for 1 h at 120 °C. Sericin solution was separated from degummed fibroin fibers and the concentration was calculated by drying of known volumes. 

### 2.4. Microsphere Preparation by Spray Drying

Five different microsphere formulations, based on algal extracts (*Chlorella vulgaris* and *Arthrospira platensis*) or silk sericin or their combination (Chl, Art, Ser, Chl-Ser, Art-Ser) were prepared. The concentration of both algal extracts and silk sericin solution was 0.8% *w*/*v*. Samples were spray dried using a Büchi Mini Spray Dryer (Flawil, Switzerland). The following process parameters were set: pump, 7 mL/min; inlet temperature, 110 °C; outlet temperature, 60 °C; air pressure, 3 bar; fluid flow, 500–600 mL/h [26,27]. Table 1 reported the relative amounts of each component. For each considered formulation, at least three batches were produced. At the end of the spray drying process, the percentage yield was determined as follows: (w_mic_/w_comp_) × 100, where w_mic_ was the weight of the spray dried microspheres and w_comp_ was the total weight of the components dissolved in the spray dried solution.

### 2.5. Microsphere Characterization

Microsphere characterization has been carried out following the current pharmaceutical requirements to qualify products and processes in an application context: particle size distribution, physicochemical characterization of solid state (morphology, Fourier transform infrared spectroscopy, Thermogravimetric Analysis), chemical composition, and, for this specific antioxidant product, ROS-scavenging activity (quality requirement). Therefore, the product cytotoxicity was assessed in an in vitro cellular model (safety requirement), and finally the wound healing potential was evaluated by a standard scratch test (efficacy requirement).

#### 2.5.1. Granulometric Analysis

Granulometric analysis of microspheres was performed by a laser light scattering granulometer (Beckman Coulter LS230, Miami, FL, USA), equipped with a small volume cell (120 mL volume; obscuration 5%); the refractive index was set at 1.359 for ethanol. A sample for each microsphere formulation was suspended in ethanol and maintained, under magnetic stirring, for 5 min; ethanol suspensions were then put into the measurement cell and ran in five replicates of 90 s each. Results were expressed as the average of at least five replicates for each microsphere formulation. The analysis of the data included the computation of the mean, standard deviation (S.D.), 10th, 50th, and 90th percentile of the volume-weighted diameter d_4,3_ (d_10_, d_50_ and d_90_, respectively) and the mean and S.D. of the surface-weighted diameter (d_3,2_). The width of the particle size distribution was described by the relative span value that was calculated as follows: (d_90_ − d_10_)/d_50_.

#### 2.5.2. Scanning Electron Microscopy (SEM)

A Zeiss EVO MA10 (Carl Zeiss, Oberkochen, Germany) was used to analyze the morphology of microparticles. The samples were gold-sputter coated under argon to render them electrically conductive prior to microscopy.

#### 2.5.3. Fourier Transform Infrared Spectroscopy (FTIR)

FT-IR spectra were obtained using a Spectrum One Perkin-Elmer spectrophotometer (Perkin Elmer, Wellesley, MA, USA) equipped with a MIRacle™ ATR device (Pike Technologies, Madison, WI, USA). The IR spectra in transmittance mode were obtained in the spectral region of 650–4000 cm^−1^ with a resolution of 4 cm^−1^.

#### 2.5.4. Simultaneous Thermogravimetric Analysis (TGA/DSC 1)

Thermogravimetric Analysis is a typical analytical technique applied in the physicochemical characterization of the solid state. In this context, it was used to quantify the water content of the products and, above all, their thermal stability, comparing the microalgae as is, and after their extraction by solvent free method and formulation in microspheres by spray-drying technique. The TGA was carried out with a Mettler STARe system (Mettler Toledo, Milan, Italy) TGA on 3–4 mg samples in 70 µL alumina crucibles (30–600 °C temperature range; heating rate 10 K min^−1^; nitrogen air atmosphere flux 50 mL min^−1^). The instrument was previously calibrated with Indium as standard reference, and measurements were carried out at least in triplicate.

#### 2.5.5. Determination of Protein Content

The protein content of each microsphere formulation was estimated by using a Micro BCA™ Protein Assay Kit (ThermoFisher Scientific, Rockford, IL, USA), according to the manufacturer’s instructions. Bicinchoninic acid (BCA) was used as detection reagent for cuprous ion (Cu^1+^) that is produced when a protein reduces Cu^2+^ in an alkaline environment. The chelation of two BCA molecules with one Cu^+^ produces a water-soluble purple complex, which exhibits a strong absorbance at 562 nm that is directly proportional to the protein content [28]. Microsphere samples were suspended in distilled water, combined with the reagents (provided with Micro BCA™ Protein Assay Kit) in a 1:1 ratio and incubated at 37 °C for 2 h. Bovine Serum Albumin (BSA), chosen as protein standard, was diluted in deionized water at different concentrations (10–75 μg/mL) and processes as reported for the samples in order to prepare a standard curve from which the protein concentration of each microsphere formulation was extrapolated. The optical density was measured at 562 nm with multi-plate reader (Synergy HT, BioTek, Swindon, UK); analyses were performed in three replicates and results were reported as μg proteins/mg sample.

#### 2.5.6. Determination of Carbohydrate Content

The carbohydrate content of each microsphere formulation was determined by using the phenol-sulfuric acid method, as reported by DuBois and colleagues [29]. Briefly, 2 mL of microspheres suspended in distilled water (0.1 mg/mL) were mixed with 1 mL of phenol aqueous solution (5% *w*/*v*) and 5 mL of sulphuric acid in test tubes. After 10 min at room temperature in dark conditions, the test tubes were placed in ice for 20 min and then centrifuged at 3000× *g* for 10 min. Glucose monohydrate, selected as carbohydrate standard, was diluted in deionized water at different concentrations (0–0.1 mg/mL) and processed in an identical manner as above in order to prepare a standard curve from which the carbohydrate concentration of each microsphere formulation was extrapolated. The absorbance was measured at 490 nm with a UV-vis spectrophotometer Uvikon 930 (Kontron Instruments, Everett, MA, USA). Analyses were performed in three replicates and results were reported as μg carbohydrates/mg sample. 

#### 2.5.7. ROS-Scavenging Activity Assay

The ROS-scavenging activity was evaluated by the DPPH (2,2-diphenyl-2-picrylhydrazyl hydrate) method, according to Chaudhuri et al., with slight modifications [30]. In detail, each microsphere formulation was tested at different concentrations (1.25, 2.5, 5.0, 10, 25 and 50 mg/mL) after dissolution in distilled water under magnetic stirring. Fifty μL of each dilution were mixed with 1950 μL of DPPH solution (1 mM) in 70% *v*/*v* methanol and kept in the dark for 20 min at room temperature. All reaction mixtures were centrifuged at 3000× *g* for 10 min and then the optical density was measured at 517 nm with a UV-vis spectrophotometer. Ascorbic acid, chosen as the positive control, was tested at the same concentrations of microsphere samples, while a reaction mixture composed by 50 μL of 70% *v*/*v* methanol and 1950 μL of DPPH solution was prepared as a negative control. The percentage of ROS-scavenging activity was calculated according to the following equation: % activity = (A − B)/A × 100, where A was the optical density of negative control and B was the optical density of the samples. Analyses were performed in three replicates, and results were reported as the mean ± standard deviation of the ROS-scavenging activity percentage.

### 2.6. In Vitro Assays

#### 2.6.1. Cytotoxicity Assay

Human fibroblast cells were seeded at a density of 10,000 cells/cm^2^ in a 96-well plate and cultured for 24 h in DMEM/F12 culture medium with the addition of 10% FBS, 1% penicillin/streptomycin and 1% amphotericin B (37 °C, 5% CO_2_). Each microsphere formulation was solubilized in the aforementioned culture medium and then filtered using 0.22 μm membranes (Merck Millipore, Tullagreen Carrigtwohill, Ireland). Different concentrations were tested: 0.05, 0.1, 0.25, 0.5, 1.25, 2.5, 5.0 and 10 mg/mL for Chl, Art and Ser formulations, while 0.1, 0.2, 0.5, 1.0, 2.5, 5.0, 10 and 20 mg/mL for Chl-Ser and Art-Ser formulations were used in order to test the cytotoxic effect of the same amount (mg/well) of algal aqueous extract and sericin. After culture medium removal, human fibroblast cells were incubated with 100 μL of each sample for 24 h and thus cell metabolic activity was evaluated by performing an MTT assay. Specifically, supernatants were discarded from each well and 100 μL of MTT solution (0.5 mg/mL) were added. After 3 h, MTT solution was substituted with 100 μL of DMSO. The optical density (OD) was measured at 570 and 670 nm (reference wavelength) with a multi-plate reader; analyses were performed in three replicates for each sample. Cell metabolic activity (%) was calculated as follows: 100 × (ODs/ODc), where ODs is the mean value of the measured optical density of each tested sample and ODc is the mean value of the measured optical density of cells incubated without microspheres (control). The study was performed in three replicates, using primary human fibroblasts obtained from 3 donors (4th passage), with viability ≥95%, (Trypan blue staining, *n* = 150), and showing no variability in term of cellular doubling time (data not shown).

#### 2.6.2. Scratch Assay

Microspheres were solubilized in the aforementioned culture medium and sterilized by filtration; different concentrations were tested: 0.05, 0.1, 0.25 and 0.5 mg/mL for Chl, Art and Ser formulations, while 0.1, 0.2, 0.5 and 1.0 mg/mL for Chl-Ser and Art-Ser formulations. 

The same three human fibroblast cell lines used in the previous experiment, seeded at a density of 40,000 cells/cm^2^ in a 24-well plate, were scraped with a sterile p200 pipet tip, creating a linear “scratch” [31]. “Scratched” cells were incubated with 1 mL of each sample. After 24, 48 and 72 h, images of treated cells were acquired using a digital camera (Lumenera Infinity 1-3C, Nepean, ON, Canada) connected to a phase-contrast microscope (Nikon Eclipse E400, Melville, New York, NY, USA) in order to evaluate the cell migration. Using scratch wounded cells and closed wound as standards, images were observed by five independent expert operators and a score between 0 (no cell migration) and 10 (completed cell migration) was given for each formulation at each considered time endpoint [27,32].

#### 2.6.3. Statistical Analysis

Raw data were processed using STATGRAPHICS XVII (Statpoint Technologies, Inc., Warrenton, VA, USA), Microsoft^®^ Excel 2013, and R, R Core Team (2014). R: A language and environment for statistical computing. R Foundation for Statistical Computing, Vienna, Austria (http://www.R-project.org). The libraries outliers by Lukasz Komsta (2011), (http://CRAN.R-project.org/package=outliersoutliers), and apl-pack by Hans Peter Wolf and Uni Bielefeld (2014), (http://CRAN.R-project.org/package=aplpack), were used to test the granulometric analysis data for the presence of univariate and bivariate outliers. A linear generalized Analysis of Variance model (ANOVA) was used to study the data. 

The amount of proteins and carbohydrates contained in each microsphere formulation was analyzed taking into account the μg proteins/mg microspheres and μg carbohydrates/mg microspheres as response variables and the microsphere formulation (Chl, Art, Ser, Chl-Ser and Art-Ser) as a fixed factor. The ROS-scavenging activity (%) was evaluated considering the effect of both microsphere formulation and concentration (1.25, 2.5, 5.0, 10, 25, 50 mg/mL). Moreover, the cytotoxicity of each formulation was analyzed, fixing microsphere formulation and concentration. The scores given by the five independent operators at each time were compared using a t-test for paired means; finally, results of scratch assay were processed, considering microsphere formulation and time (0, 24, 48 and 72 h) as fixed factors in a two-way ANOVA.

The differences between groups were analyzed with the post hoc Least Significant Difference (LSD) test for multiple comparisons. Statistical significance was set at *p* ≤ 0.05.

## 3. Results

### 3.1. Microsphere Characterization

The microalgae aqueous extracts and the sericin solution were spray dried, alone or combined, to obtain microspheres (Chl, Art, Ser, Chl-Ser and Art-Ser): the composition of the starting solutions did not influence the spray drying process, obtaining a satisfactory process yield (35 ± 8%), considering the lab-scale production.

**Figure 1:**
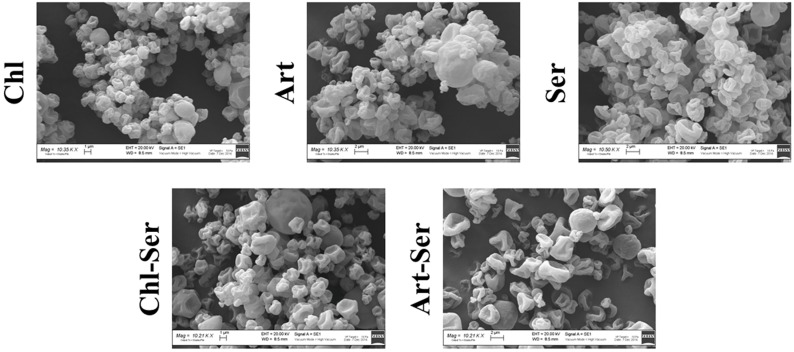
SEM images of Chl, Art, Ser, Chl-Ser and Art-Ser microsphere formulations; scale bar: 2 μm.

All formulations showed a unimodal distribution of the volume-weight (d_4,3_) and surface-weight (d_3,2_) diameters. Results evidenced that the spray drying was a suitable method to produce microspheres with similar dimensions. The 95% confidence interval (α = 0.05) of the volume-weight diameters (d_4,3_) were 4.7 ± 0.2 μm for Chl (*n* = 9), 3.5 ± 0.2 μm for Art (*n* = 10), 3.4 ± 0.1 μm for Ser (*n* = 10), 6.5 ± 0.1 μm for Chl-Ser (*n* = 5) and 4.1 ± 0.1 μm for Art-Ser (*n* = 6). The 95% confidence interval (α = 0.05) of the surface-weight diameters (d_3,2_) were 3.09 ± 0.08 μm for Chl, 2.58 ± 0.05 μm for Art, 2.1 ± 0.2 μm for Ser, 2.56 ± 0.05 μm for Chl-Ser and 2.02 ± 0.02 μm for Art-Ser. The presence of sericin elicited a slight increase of the d_4,3_ and a decrease of the d_3,2_ for Chl-Ser formulation compared with Chl and for Art-Ser compared to the Art one. The Art microspheres presented the narrowest size distribution as confirmed by the span: 1.74 for Chl, 1.37 for Art, 1.52 for Ser, 1.89 for Chl-Ser and 1.51 for Art-Ser (the values which tend to indicate a narrow distribution). Moreover, all microspheres showed typical ellipsoid or bi-concave shapes, as evidenced by the observation that the mean volume–weight diameter (d_4,3_) was always higher than the mean surface–weight diameter (d_3,2_) with high statistical significance (*p* < 0.001). These results were confirmed by the scanning electron microscopy analysis: all formulations were characterized by collapsed microspheres with a rough surface, even if some scattered smooth and round elements were appreciated in Chl-Ser and Art-Ser (Figure 1).

**Figure 2:**
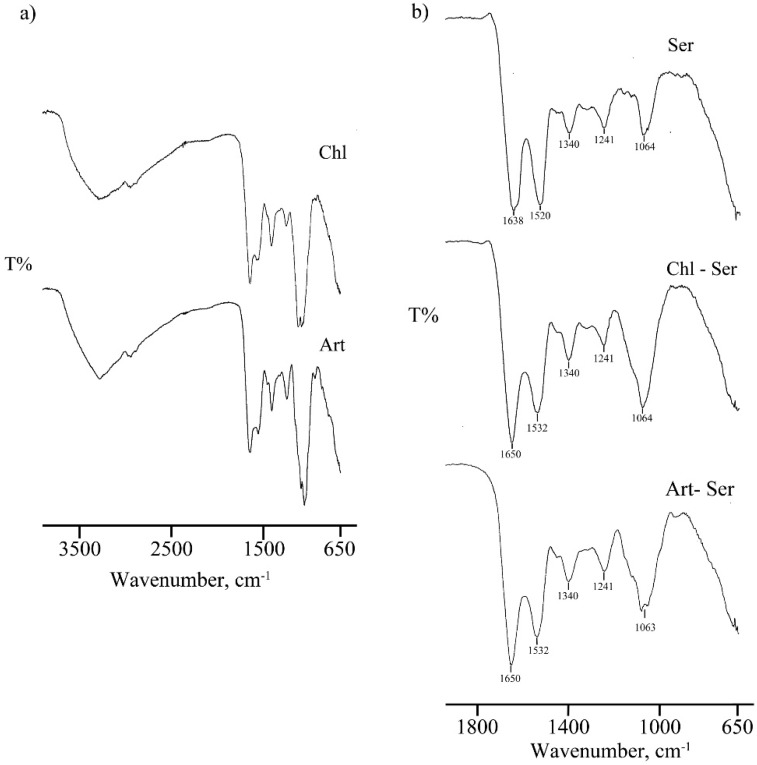
Fourier Transform Infrared Spectroscopy spectra of (**a**) Chl and Art formulations (spectral region 4000–650 cm^−1^); (**b**) Ser, Chl-Ser and Art-Ser formulations (spectral region 2000–650 cm^−1^).

The thermogravimetric analysis revealed a first mass loss of about 10% due to the absorbed water evaporation in the temperature range 30–200 °C. Furthermore, a rapid loss of weight was recorded at 220 °C indicating the decomposition of the microparticles (curves not reported). The absence of differences in the decomposition temperatures indicated that the solvent-free extraction method and spray-drying technique allowed the production of stable microspheres. In Figure 2a FT-IR spectra of Chl and Art formulations are reported: Art microspheres exhibited more intensive absorption bands, especially in the -OH phenolic region (3580–3650 cm^−1^), indicating higher antioxidant properties. The FT-IR spectra of formulations containing sericin, alone or in association with microalgae extracts, are reported in Figure 2b: Ser showed typical bands at 1638 and 1520 cm^−1^ due to amide I and II, respectively; the shift of these bands to higher wavenumbers in Chl-Ser and Art-Ser formulations is attributable to the interaction between Ser and microalgae extracts. Moreover, the band at 1399 cm^−1^ is due to symmetric deformation of -CH_2_ and -CH_3_ of proteins/carboxylic groups; finally, two typical bands, related to the stretching vibration of polysaccharides, were evidenced in the region at 1250–900 cm^−1^ (Figure 2b).

Microsphere protein and carbohydrate contents are reported in Table 2. ANOVA analyses indicated that the formulation had a significant effect on both protein and carbohydrate content (*p* < 0.001). In particular, the protein content of Ser was significantly higher than the other formulations and the simultaneous presence of silk sericin and microalgae extracts (Chl-Ser and Art-Ser) increased proteins when compared to Chl and Art (Table 2). No significant differences were observed between Chl and Art in terms of carbohydrate concentration, which showed higher content than Ser, Chl-Ser and Art-Ser (Table 2). 

### 3.2. ROS-Scavenging Activity

ANOVA analysis evidenced that both formulation and microsphere concentration had a significant effect on the ROS-scavenging activity (*p* < 0.0001).

All formulations exhibited antioxidant activity strongly correlated with the microsphere’s concentration. The equations of the straight lines obtained are presented as y = (slope ± 95% probability confidence interval)x + intercept ± 95% probability confidence interval, together with their determination coefficient (R^2^).

Art: ROS-s.a. (%) = (1.1 ± 0.1)x +7 ± 3, R^2^ = 0.992 (1)

Art-Ser: ROS-s.a. (%) = (1.11 ± 0.09)x +6 ± 2, R^2^ = 0.997 (2)

Chl-Ser: ROS-s.a. (%) = (0.96 ± 0.03)x +6 ± 1, R^2^ > 0.999 (3)

Ser: ROS-s.a. (%) = (0.6 ± 0.3)x +7 ± 8, R^2^ = 0.895 (4)

Chl: ROS-s.a. (%) = (0.66 ± 0.04)x +8 ± 1, R^2^ = 0.998 (5)

**Figure 3:**
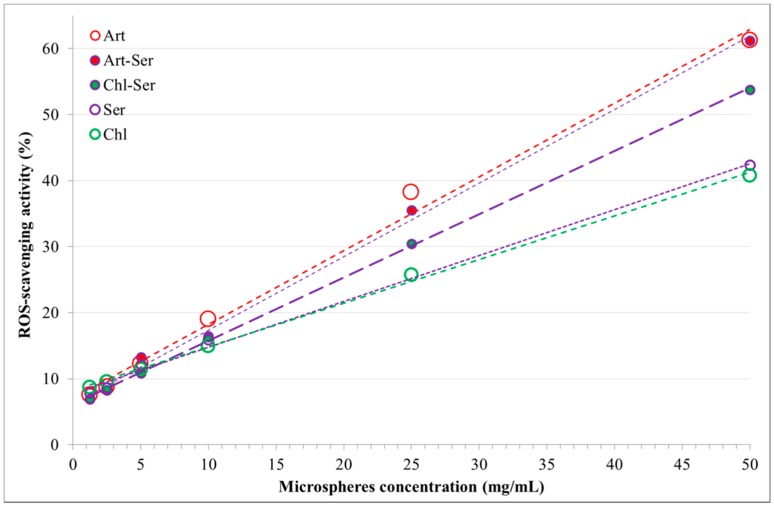
Reactive Oxygen Species (ROS)-scavenging activity (%) and microsphere concentrations (mg/mL) for the formulations studied.

Art showed the highest antioxidant property, although it was not significantly different from Art-Ser. Moreover, Art and Art-Ser formulations showed ROS-scavenging activity linear functions with slopes comparable with Chl-Ser formulation. ROS-scavenging activity of Chl-Ser was significantly higher than Chl and Ser formulations in the concentration range from 10 to 50 mg/mL. The Ser formulation was significantly more effective in preventing oxidant events than Chl, but no significant differences were highlighted with Chl-Ser. At concentrations lower than 10 mg/mL, all formulations had comparable antioxidant activity (Figure 3). 

### 3.3. In Vitro Results

**Figure 4:**
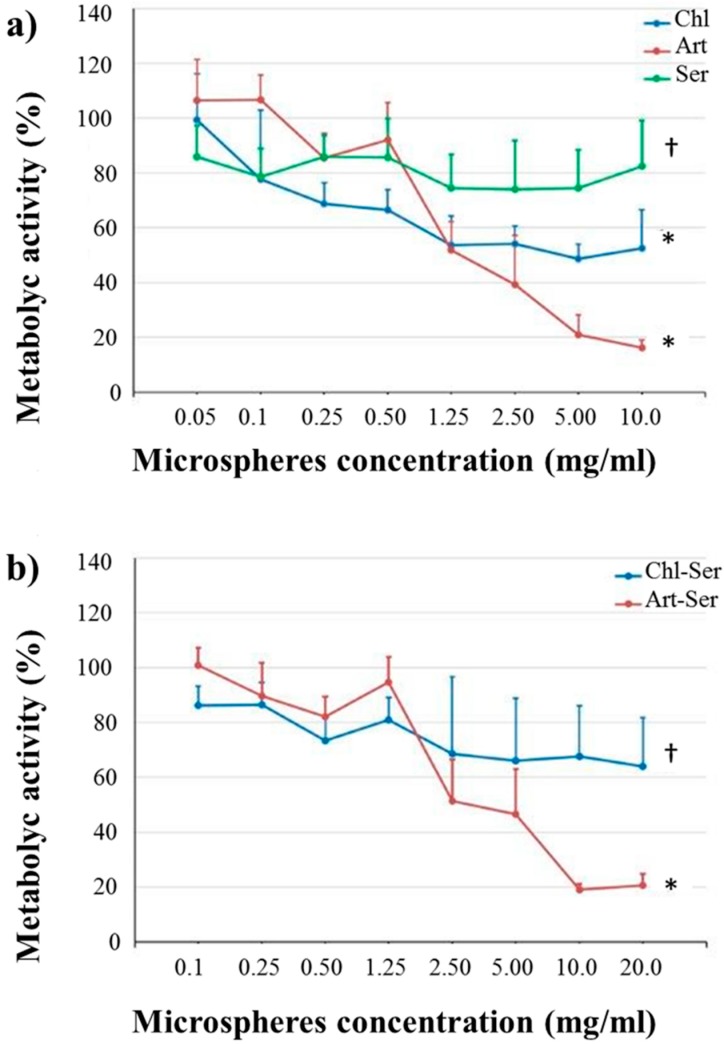
Mean values of cell metabolic activity (%) and standard deviations, considering (**a**) Chl (blue line), Art (red line) and Ser (green line) formulations and (**b**) Chl-Ser (blue line) and Art-Ser (red line) formulations. For each graph, different symbols († and *) indicate significant differences between formulations (*p* < 0.05).

A wide range of concentrations was investigated for each formulation in order to identify the non-toxic concentration of microspheres, which were further used to treat the “scratched” cells. ANOVA results showed that both formulation and microsphere concentration had a significant effect on cell metabolic activity (*p* < 0.05, Figure 4a,b).

In detail, Ser did not induce any cytotoxic effects on fibroblast cells, which showed metabolic activity higher than 75% at all tested concentrations (0.05–10 mg/mL). Conversely, both Chl and Art were found to be cytotoxic for fibroblasts starting from 1.25 mg/mL: Figure 4a shows the gradual decrease of cell metabolic activity in line with the increase of Art concentrations. Figure 4b shows the metabolic activity results for Chl-Ser and Art-Ser: the negative trend observed for Art (Figure 4a) was shown also for Art-Ser, proving that silk sericin was unable to prevent the Art cytotoxic effect. The mean % metabolic activity of cells treated with Chl-Ser was significantly higher than that of the cells treated with Art-Ser (*p* = 0.006). No significant differences between Chl-Ser and Art-Ser were observed at the lowest concentrations (0.1–1 mg/mL), with a cell metabolic activity higher than 80%. Instead, the % metabolic activity of fibroblast cells was lower than roughly 70% after treatment with Chl-Ser at 2.5–20 mg/mL and lower than 50% after treatment with Art-Ser, considering the same concentration range (Figure 4b).

**Figure 5:**
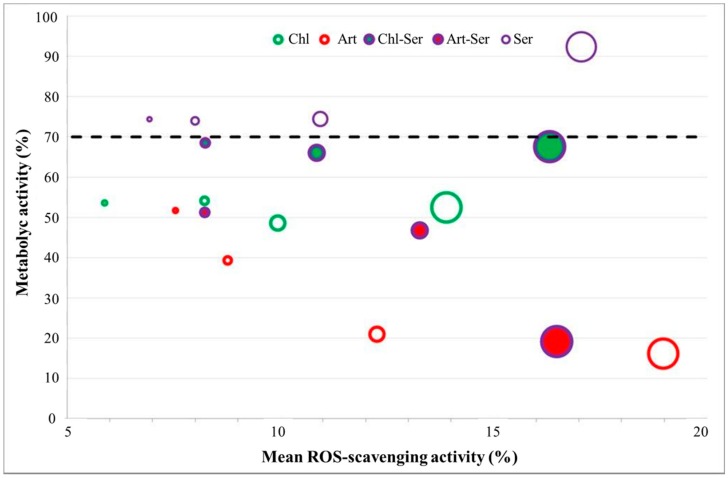
Mean ROS-scavenging activity (%) and cell metabolic activity % for the all tested formulations. The diameter of the circles in the plot is proportional to the microsphere concentration: the larger circles correspond to the 10 mg/mL, the intermediate and intermediate-small circles are those of the 5 and 2.5 mg/mL, while the smaller circles represent the properties of the 1.25 mg/mL concentration of microspheres.

Considering the cell metabolic activity together with the ROS-scavenging activity in the range of microsphere concentrations between 1.25 and 10 mg/mL, the antioxidant activity resulted in lower than 20%; the formulations which provided the better ROS-scavenging activity and the higher cell metabolic activity are those at the higher concentration of microspheres (Figure 5). As expected, Ser was cytocompatible on fibroblast cells (cell metabolic activity >70%), while the only formulation, based on silk sericin and algal aqueous extract, which provides an adequate cell metabolic activity was that obtained from *C. vulgaris* (Chl-Ser) at the concentration 10 mg/mL, offering the better compromise between antioxidant activity and cytocompatibility (Figure 5).

**Figure 6:**
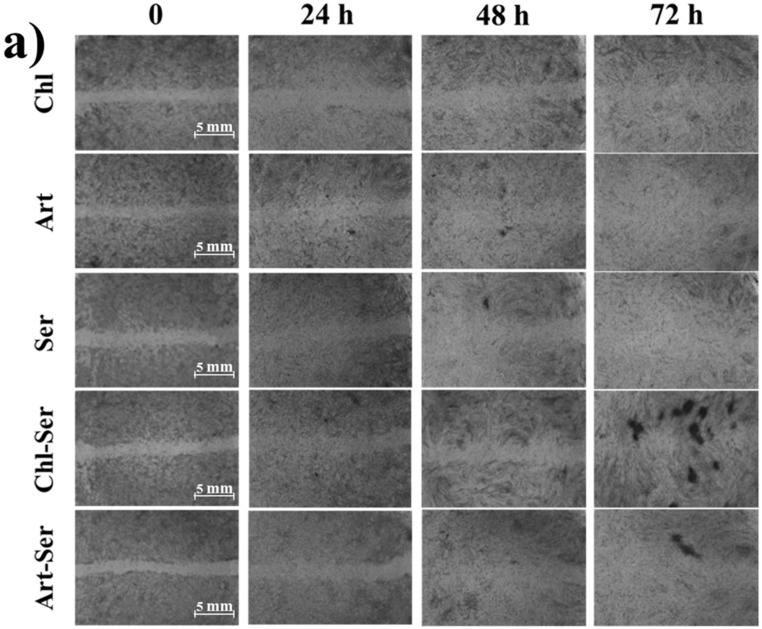

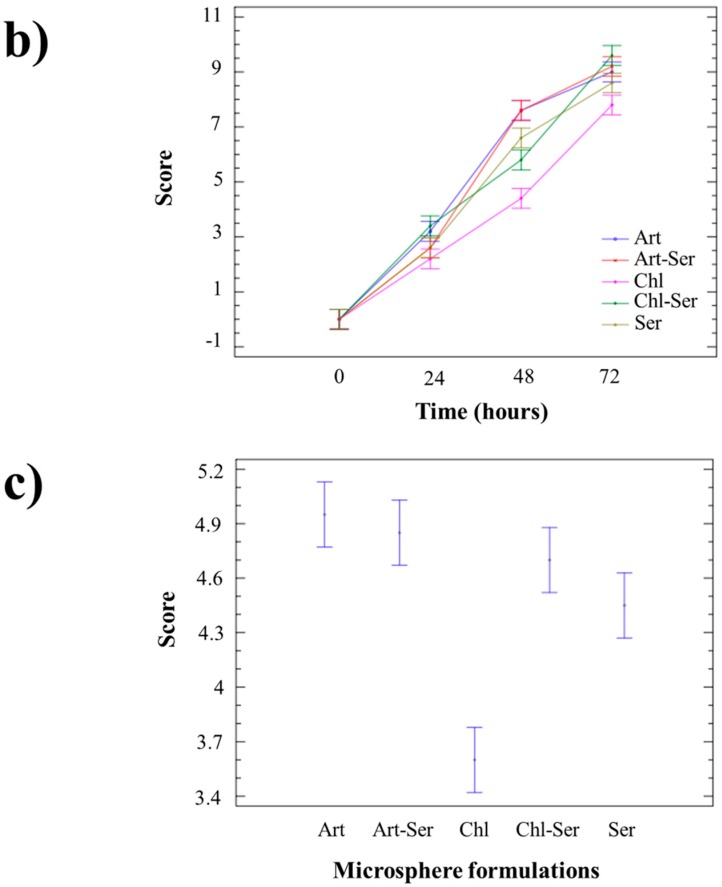
(**a**) Representative images of scratched fibroblast migration after treatment with microspheres. Mean values of wound healing scores and 95.0 percent LSD (Least Significant Difference) intervals, considering (**b**) time of treatment and (**c**) microsphere formulations.

According to these results, scratch assay was performed considering non-toxic microparticle concentrations: 0.05–0.5 mg/mL for Chl, Art and Ser and 0.1–1 mg/mL for Chl-Ser and Art-Ser. Figure 6a reports the images of cell migration after treatment with microspheres; in order to quantify the cell migration, images were observed by five independent expert operators and a score between 0 (absence of cell migration) and 10 (completed cell migration) was given for each formulation at each considered time endpoint. ANOVA results evidenced that both formulation and time significantly influenced the scores (*p* < 0.0001) (Figure 6b,c). All formulations promoted cell migration and the complete wound closure within 72 h, and this positive effect was further highlighted after treatment with Art and Art-Ser microspheres. The comparison of the mean scores obtained by the formulations of Chl, Art, and Ser showed no significant differences after 24 h (2.2 ± 1.2, 3.2 ± 1.7, and 2.6 ± 1.3, respectively), whereas at 48 h and 72 h, Chl appeared to be the worst formulation in promoting cell migration. After 48 h, the mean score ± S.D. of cells treated with Chl was 4.4 ± 0.8, and it resulted significantly lower than the mean scores of Ser, 6.6 ± 0.8, and Art, 7.6 ± 1.3 (*p* < 0.01). At 72 h, the mean score of Chl was 7.8 ± 0.2, and it resulted in scores significantly lower than the mean scores of Ser, 8.6 ± 0.3, and Art, 9.0 ± 0.0 (*p* < 0.05) (Figure 6).

Considering formulations based on silk sericin and algal extracts, the mean scores were not significantly different at 24 h (2.6 ± 0.8 and 3.4 ± 1.8, respectively) and 72 h (9.2 ± 0.2 and 9.6 ± 0.3, respectively), whereas at the 48 h time-point the Art-Ser received a mean score significantly higher than that of the Chl-Ser (7.6 ± 1.3 and 5.8 ± 0.7, respectively, *p* = 0.012) (Figure 6b).

## 4. Discussion

Microalgae represent a novel natural source of highly interesting bioactive compounds: recently, their use has been considered in tissue regeneration, developing innovative scaffolds such as nanofibers based on polyethylene oxide [33], polycaprolactone [34] and silk fibroin [35]. 

For the first time, in this work, *C. vulgaris* and *A. platensis* aqueous extracts have been used in combination with silk sericin, obtained from *Bombyx mori* cocoons, for the production of microspheres suitable for the topical treatment of skin wounds. Specifically, *C. vulgaris* and *A. platensis* aqueous extracts and silk sericin were spray dried, alone or combined, to obtain five microsphere formulations (Chl, Art, Ser, Chl-Ser and Art-Ser). The aqueous extraction of both microalgae was performed to obtain a pool of phytochemicals that have synergistic or additive effects as reported in literature [25], particularly against the oxidative stress. Sericin, due to its wound healing potential [18], may improve the regenerative effect of the microalgae extracts; moreover, it represents an adequate polymeric carrier [36] for the microencapsulation process of algal extracts. Sericin microspheres loaded with naringenin have previously been developed in our group for the topical treatment of middle-stage psoriasis, highlighting that the microencapsulated drug was more effective in the down regulation of TNF-α than as a free drug [24]. 

Even though *Arthrospira platensis* and *Chlorella vulgaris* are approved by FDA as GRAS, for safety requirements, our formulations were tested in vitro on fibroblast at different concentrations. According to our results, all the formulations showed good cytocompatibility: Ser did not induce any cytotoxic effect on fibroblast cells; while Art and Chl started to be cytotoxic at the higher concentrations. In this regard, sericin was unable to reduce the cytotoxic effect of Art.

Subsequently, wound healing potential was investigated for all the formulations at the non-toxic microparticle concentrations. The in vitro scratch assay is an easy method suitable to study the effects of cell–matrix and cell–cell interactions on cell migration and to mimic cell migration during wound healing in vivo. The scratch assay has revealed that all the formulation tested promoted cell migration and a complete wound closure within 72 h. In particular, the wound healing potential of *A. platensis* extract seemed to be higher than *C. vulgaris*. The addition of Ser to Chl formulation improve the ability to promote wound closure, while Art-Ser formulation did not result more effective than Art. Our results are not in contrast with literature: due to its mitogenic effect, sericin was recently reported to support keratinocyte and fibroblast adhesion and proliferation, promoting collagen deposition in damaged areas and thereby accelerating wound re-epithelialization [37]. In the literature, the wound healing potential of *C. vulgaris* and *A. platensis* has been less discussed. To the best of our knowledge, there have been very few papers published investigating the use of microalgae in wound healing. Syarina et al. [38] have demonstrated that *A. platensis* was effective in promoting the wound healing process: the phytochemical profile of microalgae aqueous extract was defined, showing the presence of several therapeutic compounds useful in chronic wound treatment. *C. vulgaris* has also been shown to be a potential source of bioactive agents capable of accelerating the wound healing process with minimal scar formation, as confirmed by Zailan et al. [39].

The use of spray dried microparticulate systems has generated great attention in skin regeneration: microspheres are adequate systems for drug encapsulation and for controlling the release of active molecules, depending on physicochemical properties of polymers, even if other formulations are quite common as creams, bandages, films, sponges and ointments [40]. There may be many reasons for the formulation of antioxidant algal extract in microspheres, mainly related to their intrinsic lability: isolation of active ingredients from external environment, moisture protection, oxidation protection, light exposure protection, aroma masking, and metering of ingredients. Moreover, the algal extracts in its native liquid form have disadvantages in portability and commercial viability for use as natural antioxidant sources: microencapsulation of the algal extracts in silk sericin would circumvent these aspects, albeit it does not provide sustained/prolonged/slow release of active, since sericin is a water-soluble protein.

Biodegradable polymers are preferred in wound healing due to their surface properties and their biocompatibility [41]. The main advantage of microparticulate systems is related to their easy fabrication. Spray drying is an economically feasible technique that has already been used to produce dry biomass powders enriched with bioactive molecules without altering their physicochemical and, hence, functional properties [42]. In the last decades, some research groups have evaluated the effect of spray drying on the biological content of different algal species. In particular, Leach et al. and Orset et al. [43,44] have investigated the β-carotene content and stereoisomer composition of spray dried *Dunaliella salina* biomass: the spray-drying process neither promoted an excessive degradation nor a change in isomer composition of β-carotene. Furthermore, Leach et al., [43] evidenced that the stability of the antioxidant compound greatly improved when *D. salina* cells were microencapsulated in a polymeric blend of maltodextrin and gum arabic, minimizing β-carotene degradation in the presence of light and oxygen. 

Granulometric analysis, performed by laser light scattering, and SEM images evidenced that spray drying was a robust technique which allowed the obtainment of microspheres with dimensions and morphology suitable for the purposes of the present study.

A wrinkled texture characterized the surface of all microsphere formulations, even if some scattered smooth and round particles were appreciated in the presence of sericin. As reported by Mishara et al., [45], microsphere morphology may be influenced by irregular shrinkage forces during the spray drying process, which could be related to the formulation composition and process parameters. The solvent vapor pressure and the polymer concentration might be critical factors, able to condition particle morphology, as reported by Raula et al., [46]. Genç et al., [47] have observed that the spherical structure of sericin spray dried particles tend to collapse due to fast solvent evaporation, which is strictly correlated to the low concentration of the starting sericin solution and the lack of excipients; the same morphological results were observed by Chlapanidas et al., [22]. The rough appearance that characterized all formulations might be also explained by the collapse of microparticles during SEM analysis, performed according to the sputtering technique.

Proteolytic enzymes, proinflammatory cytokines, growth factors and ROS are secreted by cells with a crucial role during the wound healing process, albeit that high concentrations of these substances can alter the wound re-epithelialization through the degradation of extracellular matrix and the functionality of fibroblasts and keratinocytes. Antioxidant compounds are able to neutralize the deleterious effect of ROS [48]. Several publications reported in the literature have attributed the antioxidant potential of algal biomass mainly to their phenolic content, although many other compounds, such as amino acids and polysaccharides, are also known to be antioxidants [49]. The composition of *C. vulgaris* and *A. platensis* extracts was recently examined by other authors in order to identify the compounds responsible for their antioxidant potential [15]. Our results evidenced that Ser had more protein and less polysaccharides than algal aqueous extract. Art formulation was significantly richer in proteins than Chl, while both Art and Chl microspheres were constituted by about 10% *w*/*w* of polysaccharides. Moreover, the protein content of each microsphere formulation was related to its theoretical composition: a detailed analysis demonstrated that total proteins contained in Art-Ser resulted as the half algebraic sum of proteins contained in Ser and Art. An analogous assessment was carried out for *C. vulgaris* and for the carbohydrate content. The higher protein content of Art (compared to Chl) and the higher polysaccharides content (compared to Ser) may be responsible to its more promising biological effects, especially regarding the antioxidant activity.

According to DPPH results and FTIR analyses, the *A. platensis* aqueous extract and, to a significantly lesser extent, the silk sericin were the most responsible for the microsphere ROS-scavenging activity. These results were confirmed by Wu et al. [50], who compared *A. platensis* and *C. vulgaris* aqueous extracts in terms of antioxidant and anti-proliferative properties. They highlighted a correlation between the antioxidant activity and the extract composition, particularly with the total phenolic content. *A. platensis* extract was a stronger antioxidant than *C. vulgaris*: total phenolic compounds extracted with water from *A. platensis* were almost five times more than those contained in *C. vulgaris* [50].

The ROS-scavenging activity of sericin has been extensively investigated both in vitro and in vivo; it has been explained due to the high presence of hydroxyl amino groups in its amino acid sequence, mainly from serine and threonine [21]. Moreover, silk sericin has been shown to protect human fibroblasts against oxidative damage when used in sponge-like dressings intended for the treatment of chronic skin ulcers [19]. 

In conclusion, *Arthrospira platensis* aqueous extract showed the highest antioxidant activity and the capability to induce a complete wound closure within 72 h. In contrast to our hypothesis, the combination of *Arthrospira platensis* aqueous extract and sericin did not prove more effective with respect to algal extract alone; this could be probably due to algal high biological activity, which conceals the silk sericin effect. The microencapsulation process may not be a confounding factor for the combination studied: sericin is a soluble polymer and thus there is no risk for a slow release of active algal. Silk sericin can be considered an optimal polymeric carrier for phytocomplex microencapsulation process, and a murine in vivo model of wound healing will consolidate the in vitro efficacy results obtained in this work.

## Figures and Tables

**Table 1:** Theoretical composition of microspheres reported as % *w*/*w* of *A. platensis* extract, *C. vulgaris* extract and silk sericin in each formulation (Chl, Art, Ser, Chl-Ser and Art-Ser).

Formulation	*A. platensis* Extract	*C. vulgaris* Extract	Silk Sericin
Chl	0	100	0
Art	100	0	0
Ser	0	0	100
Chl-Ser	0	50	50
Art-Ser	50	0	50

**Table 2:** Protein and carbohydrate contents in the resulting microspheres. Results are reported as mean values ± S.D. (*n* = 3). Different letters (a, b, c, d and e) indicate significant differences between formulations (*p* < 0.0001).

Formulation	Protein Content (μg Proteins/mg Microspheres)	Carbohydrate Content (μg Carbohydrates/mg Microspheres)
Chl	162.74 ± 2.11 ^a^	97.45 ± 3.29 ^a^
Art	217.41 ± 21.26 ^b^	100.50 ± 6.67 ^a^
caSer	1104.89 ± 46.07 ^c^	17.14 ± 0.05 ^b^
Chl-Ser	640.94 ± 8.02 ^d^	46.48 ± 0.10 ^c^
Art-Ser	714.35 ± 21.95 ^e^	36.53 ± 3.45 ^d^

## Abbreviations

ANOVA	Analysis of variance
BCA	Bicinchoninic acid
BSA	Bovine serum albumin
DMSO	Dimethyl sulfoxide
DPPH	2,2-Diphenyl-2-picrylhydrazyl hydrate
FTIR	Fourier transform infrared spectroscopy
GRAS	Generally recognized as safe
MTT	3-(4,5-Dimethylthiazol-2-yl)-2,5-diphenyltetrazolium bromide
ROS	Reactive oxygen species
SEM	Scanning electron microscopy
TGA	Thermogravimetric Analysis
